# Influence of Hexylene Glycol Terephthalate Chain Segments on the Crystallization and Thermal Properties of Polyamide 6

**DOI:** 10.3390/polym17121687

**Published:** 2025-06-17

**Authors:** Zeyang Li, Qiang Ren, Shan Mei, Wei Liu, Guangyi Zhou, Baoning Zong

**Affiliations:** 1 No. 22 Research Department, Research Institute of Petroleum Progressing, SINOPEC, Beijing 100083, China; lizeyang.ripp@sinopec.com (Z.L.); meishan.ripp@sinopec.com (S.M.); liuwei2.ripp@sinopec.com (W.L.); zhouguangyi.ripp@sinopec.com (G.Z.); 2 Basic Research Department, Research Institute of Petroleum Progressing, SINOPEC, Beijing 100083, China; renqiang.ripp@sinopec.com; 3 State Key Laboratory of Catalytic Material and Reaction Engineering, Research Institute of Petroleum Progressing, SINOPEC, Beijing 100083, China

**Keywords:** copolymer PA6, polyesteramide, thermal properties, crystallization behavior, molecular simulation

## Abstract

In this study, a poly [ε-caprolactam-*co*-bis(2-hydroxyethyl) terephthalate] copolymer (P (CL-*co*-BHET)) was synthesized from para-terephthalic acid (PTA), ethylene glycol (EG), and caprolactam (CL). The crystallization behavior and thermal stability of the copolymer were thoroughly investigated. With the aid of molecular simulation, this study investigated the variation in interchain hydrogen bonding in the copolymer, focusing on the direction and the number of hydrogen bonds. The results revealed a close relationship between the copolymer chain structure, the variation in interchain hydrogen bonding, and the crystallization behavior and thermal stability of the copolymer. The introduction of BHET segments disrupted the regularity of the PA6 backbone and hydrogen bonding, leading to a decrease in the melting point, crystallization temperature, and crystallinity of the copolymer. The thermal stability of the copolymers also decreased, and the crystallization form gradually shifted from the α-crystalline to the γ-crystalline phase. The findings of this study are significant for evaluating the crystallization behavior of polyester amides and for predicting and regulating the properties of polyesteramide polymers.

## 1. Introduction

Polyamide, commonly referred to as nylon, is a polymer characterized by amide bonds. Among various types of polyamide, polyamide 6 (PA6) is the most extensively produced. It is typically synthesized via the ring-opening polymerization of caprolactam. The intermolecular hydrogen bonding in PA6 results in strong intermolecular forces and a regular molecular structure. As a semi-crystalline polymer, PA6 exhibits favorable thermal stability and mechanical properties, making it suitable for applications in engineering plastics, films, and fibers [1,2,3,4,5]. Polyesters represent a class of polymers connected by ester bonds, which are known for their excellent hydrolytic stability and biocompatibility [6]. Polyester amide, an advanced material derived from the copolymerization of alternating ester and amide chain segments, integrates the advantages of both polyamides and polyesters. The copolymerization with polyester serves as a significant approach for modifying polyamides. This method not only retains the superior mechanical properties of polyamides but also endows them with enhanced thermal properties, biocompatibility, and biodegradability [7]. Consequently, polyester amides have broader application prospects compared to polyamides and can be utilized in hot-melt adhesives [8], foams [9], biodegradable materials [10,11], etc.

Recent investigations into polyester amides have revealed that random copolymerization typically leads to a reduction in the melting temperature and concurrently impacts the crystallization behavior [12,13]. The crystallization mechanisms of polyamides, particularly PA6, have been extensively elucidated. PA6 exhibits two primary stable crystalline forms: α-crystalline and γ-crystalline. The α-crystalline form is thermodynamically favored, whereas the γ-crystalline form is kinetically favored. The formation of these two crystalline types in PA6 is attributed to distinct hydrogen bond arrangements, and they can interconvert under specific conditions. For instance, annealing, high-temperature crystallization, and solution crystallization promote the formation of the α-crystalline form, while rapid cooling from the melt and high-speed spinning favor the γ-crystalline form [14,15,16,17]. However, the crystallization behavior of polyesteramide copolymers warrants further attention, as it is crucial for understanding and predicting material properties. Evaluating the crystallization behavior to optimize processing conditions is highly advantageous for controlling polymer stability and properties, as well as for the development of final products [18]. Current research often focuses on polyesteramide synthesis, but a comprehensive study that explores the underlying mechanisms of how BHET segments influence hydrogen bonding and induce crystalline phase transformations is lacking. This limits our ability to predict and tailor the properties of polyester amide copolymers for specific applications. To address this, our study specifically investigates the interplay between BHET-induced hydrogen bonding rearrangement and crystalline phase transformation, highlighting the mechanisms through which BHET segments influence the crystallization behavior of the copolymers.

In this study, the poly[ε-caprolactam-*co*-bis(2-hydroxyethyl) terephthalate] copolymer (P(CL-*co*-BHET)) was synthesized using para-terephthalic acid (PTA), ethylene glycol (EG), and caprolactam (CL). The crystallization properties and thermal stability of the copolymer were thoroughly investigated. Molecular simulations were employed to examine the changes in the interchain hydrogen bonding of the copolymer molecules, specifically focusing on the influence of the main chain angle and the number of hydrogen bonds. This study elucidated the relationship between the copolymer molecular chain structure, the changes in interchain hydrogen bonding, the crystallization behavior, and the thermal stability of the copolymer. These findings not only provide a deeper understanding of the structure–property relationships in polyester amides but also offer valuable insights for the development of new materials with tailored properties for specific applications.

## 2. Experimental Section

### 2.1. Materials

ε-Caprolactam (CL) was supplied by Sinopec Hunan Petrochemical (Hunan, Yueyang City, China). Para-terephthalic acid (PTA), ethylene glycol (EG), 2,2,2-trifluoroethanol, and deuterotrifluoroacetic acid were purchased from Acros (Beijing, China). All the above reagents are of analytical grade and used without further purification.

### 2.2. Synthesis of BHET

PTA (46.2 g) and EG (25.9 g) were mixed to form a slurry. The slurry, along with antimony ethylate (0.017 g) and sodium acetate (0.005 g), was then added to a 250 mL reactor at room temperature. The air inside the reactor was removed under vacuum, and the reactor was subsequently purged with dry, high-purity nitrogen (N_2_). This vacuum-purging operation was repeated three times to ensure the removal of residual air. The temperature was then raised to 240 °C, and the reaction was maintained at this temperature for 2 h with the pressure controlled at 0.15 MPa and the stirring speed set at 100 rpm. During this period, water was slowly removed from the system, with approximately 98% of the theoretical water amount being expelled. At the end of the reaction, a white powder of bis(2-hydroxyethyl) terephthalate oligomer (BHET) was obtained. This oligomer was repeatedly washed with deionized water and dried under vacuum at 60 °C.

### 2.3. Copolymerization of CL and BHET

The dried BHET and CL were added to the 250 mL reactor in a predetermined ratio. Water (3 wt%) was introduced as a catalyst. The reactor was then evacuated at room temperature to remove air, and dry, high-purity nitrogen (N_2_) was introduced. This vacuum-purging operation with N_2_ was repeated three times to ensure the removal of residual air. Subsequently, dry high-purity N_2_ was continuously introduced until the pressure inside the reactor reached approximately 0.1 MPa. The reaction was conducted at 200 °C for 1 h and then at 260 °C for 5 h. Finally, the system was evacuated to −0.05 MPa, and continued to react for 1.5 h. At the conclusion of the reaction, the copolymer was extruded from the reactor in a molten state and subsequently cooled to yield the polyesteramide copolymer P(CL-*co*-BHET).

### 2.4. Characterization

The chemical structures of the polymers were investigated by ^1^H NMR (Bruker AVANCE NEO 500 MHz, Rheinstetten, Germany), using trifluoroacetic acid-d as the solvent.

The thermal properties of PA6 samples were tested using a Differential Scanning Calorimeter (DSC, DSC3 METLER TOLEDO, Greifensee, Switzerland) at temperatures ranging from 25 to 260 °C. The testing process consisted of two ramp-ups and one cool-down, with the first ramp-up eliminating the thermal history, and the rate of the primary cool-down versus the secondary ramp-ups was 10 °C/min. The tests were carried out in aluminum pans under a nitrogen atmosphere. The degree of crystallinity (*X_c_*) was calculated by the following equation:

(1) $$X_{C}\left(\%\right)=\frac{∆H_{m}}{∆H_{100}}\times 100\%$$

where ∆*H_m_* is the specific enthalpy of melting, and ∆*H*_100_ is the enthalpy of melting with 100% crystalline nylon 6 (188 J/g) [19].

The crystalline structures of the PA6 samples were tested using an X-ray diffractometer (XRD Empyrean, Almelo, The Netherlands) with a radiation source of Cu-K α-rays (λ = 0.15406 nm), a scanning rate of 2°/min, a scanning area ranging from 5° to 35°, a voltage of 40 kV, and a current of 30 mA.

The thermal stability properties of the PA6 samples were tested using a thermogravimetric analyzer (TGA, TGA2 METLER TOLEDO, Greifensee, Switzerland). The tests were carried out in a ceramic crucible under an N_2_ atmosphere at temperatures ranging from 25 °C to 600 °C with a temperature increase rate of 10 °C/min.

### 2.5. Molecular Simulation

The molecular simulations were conducted using an IBM FLEX X240 clustered server, employing the Material Studio 2017R2 software. Molecular dynamics optimization was performed using the NVE system with the Andersen method for temperature control at 600 K. The Compass force field was utilized with a time step of 1 fs and a total simulation time of 500 ps. Following this initial relaxation phase, the molecular dynamics simulation was continued at 300 K for an additional 1000 ps using the NPT system to refine the structure and analyze the relevant properties. The simulation outputted one structure for every 500 steps of the calculation, with an intercept radius of 1.35 nm.

## 3. Results and Discussions

**Figure 1:**
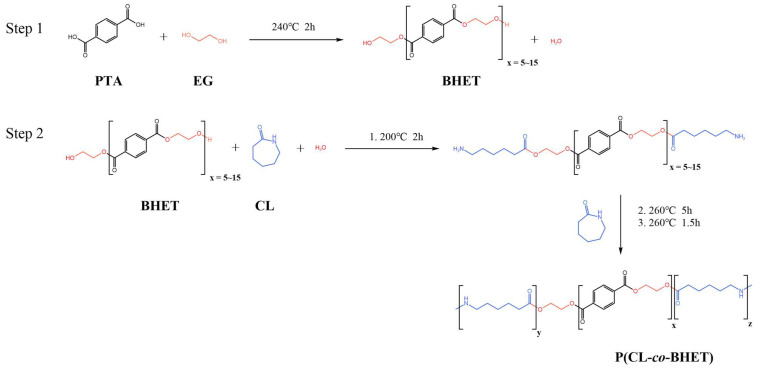
Preparation process of P(CL-*co*-BHET).

The PTA and EG were first esterified to produce low-molecular-weight BHET, which exhibited a degree of polymerization within 5 to 15, with an average polymerization degree of 10. Thereafter, the BHET was subjected to copolymerization with caprolactam and water in diverse molar ratios. This copolymerization procedure resulted in the formation of a series of polyester amide copolymers, designated as P(CL-*co*-BHET). The detailed reaction process is illustrated in Figure 1.

**Figure 2:**
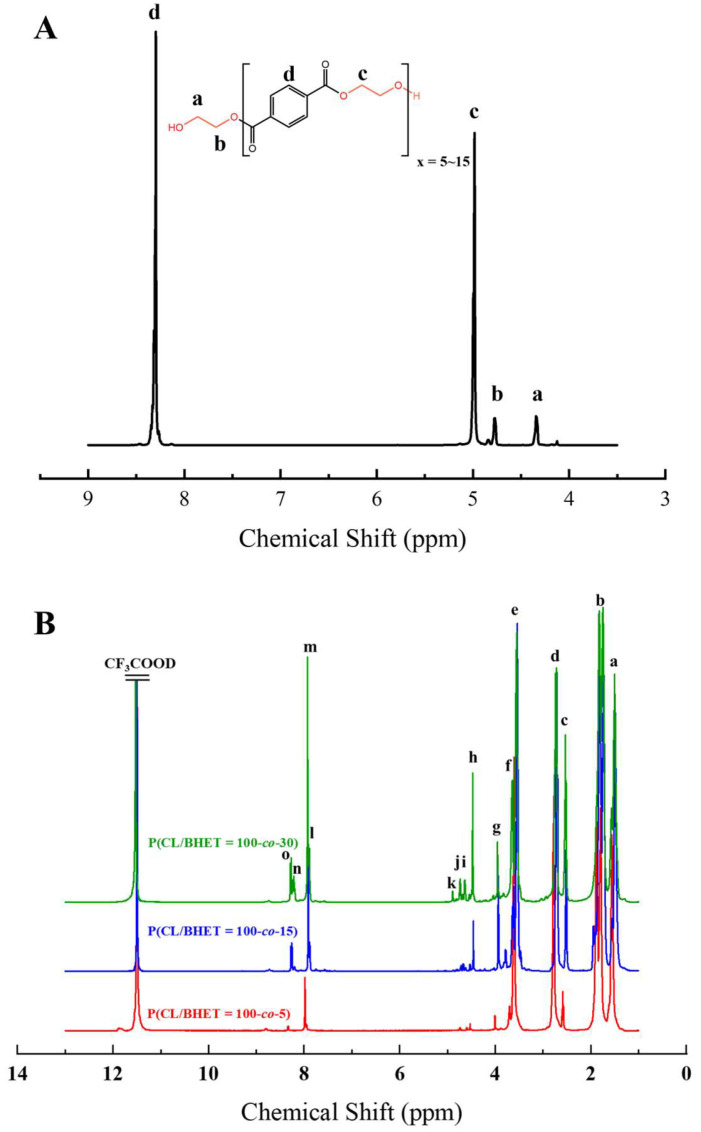
The ^1^H NMR s of BHET (**A**) and P(CL-*co*-BHET) (**B**).

The structure of the polymer was elucidated using ^1^H NMR spectroscopy. Figure 2A presents the ^1^H NMR spectrum of the BHET, where the chemical shift peaks are as follows: δ4.34 (a) and δ4.78 (b) correspond to the hydrogen atoms on the methylene groups at the terminal ends, while δ4.98 (c) represents the hydrogen atoms on the methylene groups within the main chain of the BHET. The peak at δ8.30 (d) is attributed to the hydrogen atoms on the benzene ring. Based on the area ratio of the c peak to the a peak, the average degree of polymerization of the BHET is determined to be approximately 10. Figure 2B displays the ^1^H NMR spectrum of the copolymer P(CL-*co*-BHET). The copolymerization product is relatively complex due to the ester–amide bond exchange that occurs during the copolymerization process [20]. This bond exchange results in a more homogeneous distribution of the BHET chain segments within the P(CL) molecular chain. The chemical shift signals observed in the ^1^H NMR spectra provide detailed insights into the structural composition of the copolymer. The signals at δ1.50 (a), δ1.74, δ1.83 (b), δ2.72 (d), and δ3.55 (e) correspond to hydrogen atoms on the P(CL) main chain, while the signal at δ2.53 (c) corresponds to hydrogen atoms on the methylene group attached to the -C=O- of the ester bond. The signal at δ3.64 (f) corresponds to hydrogen atoms on the methylene group attached to the -NH- of the amide bond on the side of the benzene ring. The signals at δ3.95 (g), δ4.47 (h), δ4.64 (i), δ4.73 (j), and δ4.89 (k) correspond to hydrogen atoms on the two methylene groups of EG, with different signals indicating different bonding configurations. The signals at δ7.90 (l), δ7.93 (m), δ8.21 (n), and δ8.27 (o) correspond to hydrogen atoms on the benzene ring of PTA, with different positions indicating different bonding environments. These detailed chemical shifts help to elucidate the various possible structures of the copolymer, as listed in Table 1.

**Figure 3:**
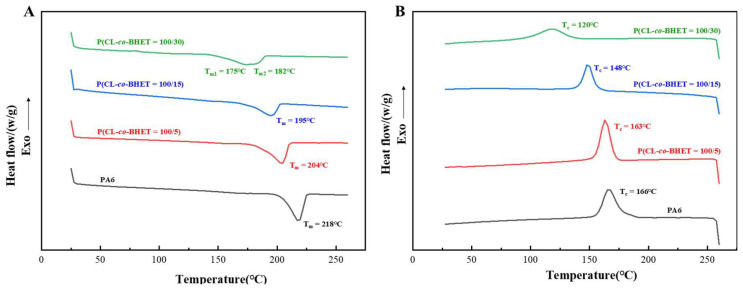
(**A**): Melting curves of PA6 and P(CL-*co*-BHET) copolymers with different BHET polymerization content; (**B**): crystallization curves of P(CL-*co*-BHET) copolymers with different BHET polymerization content.

The melting and crystallization behaviors of the PA6 and P(CL-*co*-BHET) copolymers are illustrated in Figure 3. As the BHET content increases, the melting and crystallization temperatures of the copolymers gradually decrease, and the melting peaks become progressively broader. Specifically, when the BHET content rises from 0% to 30%, the melting point of the polymer drops from 218 °C to 175 °C, the crystallization temperature decreases from 166 °C to 120 °C, and the crystallinity reduces from 25.4% to 15.5% (Table 2). These changes are attributed to the disruption of the regularity of the PA6 molecular chain and hydrogen bonding by the incorporation of BHET segments. The presence of BHET segments hinders the close stacking of PA6 molecular chains during crystallization, thereby reducing the stability of the crystal structure and leading to a lower melting point. The rigid structure of BHET segments restricts the motion of PA6 during crystallization, resulting in a lower crystallization temperature. The addition of BHET also reduces the number of hydrogen bonds, weakens the chain regularity during crystallization, and thus lowers the degree of crystallinity. Notably, when the BHET content reaches 30%, the melting peak exhibits a tendency to split into two peaks. This may be due to the formation of a certain length of block structure in the molecular chain, leading to slight phase separation, where PBHET and P(CL-*co*-BHET) segments crystallize separately and show two distinct melting points. This phenomenon is also supported by the ¹H NMR characterization results of P(CL-*co*-BHET = 100/30) and has been similarly observed in studies of poly[(ε-caprolactone)-(ω-aminoundecanoic acid)] copolymers [21].

**Figure 4:**
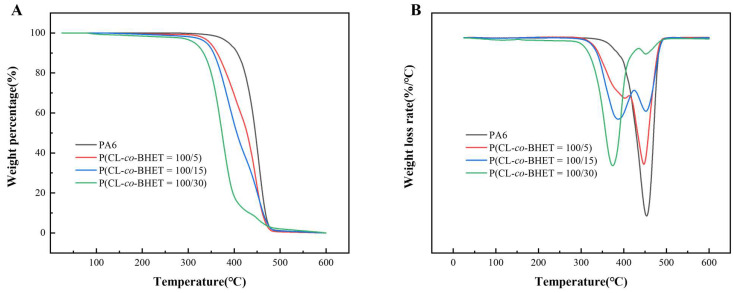
(**A**): The TGA curves of PA6 and P(CL-*co*-BHET) copolymers with different BHET polymerization content; (**B**): the DTA curves of PA6 and P(CL-*co*-BHET) copolymers with different BHET polymerization content.

The TGA/DTA curves under a nitrogen atmosphere (Figure 4) show the thermal degradation behavior of the PA6 and P(CL-*co*-BHET) copolymers. The thermal decomposition temperature (T_d5%_) at a mass loss of 5 wt% decreases with increasing BHET content. This trend is consistent with observations in other copolymer systems such as poly[(ε-caprolactam)-(ε-caprolactone)] copolymers [22]. In contrast to the single-stage degradation observed in pure PA6, the P(CL-*co*-BHET) copolymers exhibit a two-stage degradation pattern characterized by a maximum thermal decomposition rate at two different temperatures (T_f_). As the BHET content increases, T_f1_ gradually decreases, and the proportion of the first-stage thermal weight-loss component increases, as evidenced by a gradual decrease in the residual mass percentage (W_1_) at the maximum rate of the first thermal decomposition. Meanwhile, T_f2_ remains relatively constant, but the residual mass percentage (W_2_) at T_f2_ also decreases. Detailed thermal stability data are shown in Table 3, where T_d5%_ decreases from 386 °C to 315 °C, W_1_ decreases to 48.5%, and W_2_ decreases to 10.0% as the BHET content reaches 30%. Despite these changes, the T_d5%_ of all the P(CL-*co*-BHET) copolymers remains above 310 °C, indicating that these copolymers are thermally stable enough for normal processing and application. The first stage of degradation is primarily attributed to the thermal degradation of the P(BHET) chain segments, with a higher BHET content leading to faster mass loss. The second stage corresponds to the thermal degradation of the P(CL) chain segments. The degradation mechanism of P(CL-*co*-BHET) copolymers is complex. The mass loss at each stage is not equivalent to the corresponding mass content of the P(BHET) and P(CL) chain segments. The degradation products of the P(BHET) chain segments may influence the thermal degradation of the P(CL) chain segments, and the interactions and synergistic effects between different chain segments also affect the overall thermal degradation process of the copolymers.

**Figure 5:**
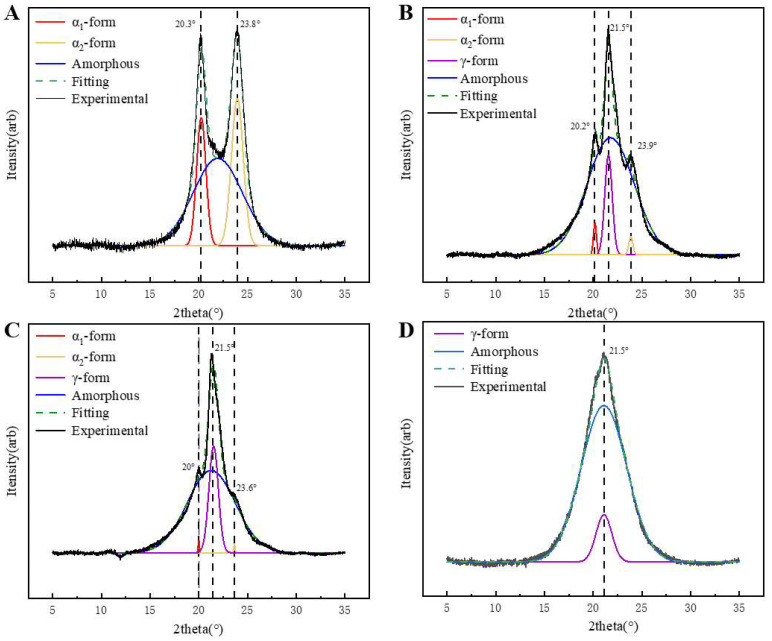
The wide-angle X-ray diffraction (WAXD) profiles of the (**A**): pure polyamide 6; (**B**): P(CL-*co*-BHET = 100/5); (**C**): P(CL-*co*-BHET = 100/15); (**D**): P(CL-*co*-BHET = 100/30).

The XRD diffraction patterns of PA6 and P(CL-*co*-BHET) copolymers are presented in Figure 5. Figure 5A shows the XRD pattern of pure PA6, which exhibits two characteristic diffraction peaks at 2θ = 20.1° (200) and 23.8° (002, 202), corresponding to the α-crystalline form [23]. Upon the addition of BHET, the α-crystalline form gradually transforms into the γ-crystalline form. When the polymerized content of BHET is 5%, the copolymer displays both the α- and γ-crystalline forms, with an additional characteristic peak at 2θ = 21.5° (100) (Figure 5B), indicative of the γ-crystalline form. As the BHET content increases to 15%, the copolymer continues to exhibit the coexistence of the α- and γ-crystalline phases, but the proportion of the α-crystalline form decreases, with the γ-crystalline form becoming dominant (Figure 5C). When the BHET content reaches 30%, the copolymer crystals are entirely γ-crystalline (Figure 5D). This transformation is attributed to the changes in the hydrogen bonding arrangement caused by BHET, including the number of hydrogen bonds and the deflection angle between hydrogen bonds and the main chain, which promotes the formation of the γ-crystalline form. And the stable arrangement of the methylene chain can make the γ-crystalline type more stable [24,25]. In addition, the Scherrer equation (Equation (2)) was used to determine the crystal size (*L_HKL_*) to study the relationship between the crystallization behavior of the P(CL-*co*-BHET) copolymers and the crystal size.

(2) $$L_{HKL}=\frac{K\lambda }{\beta \;\cos\theta }$$

where *K* is the shape factor, taken as 0.89 for all peaks, and *β* is the full width at half peak (FWHM) of the diffraction peaks with radian (i.e., FWHM × π/180). The calculated microcrystalline parameters of P(CL-*co*-BHET) copolymers are shown in Table 4. With the increase in BHET content, the grain size of the α-crystalline type gradually increases, while its proportion decreases. Conversely, the grain size of the γ-crystalline type decreases, and its proportion increases. The crystallization behavior of PA6 can be attributed to the inherent regularity of its molecular chains, which promotes an orderly crystallization process. This regularity leads to more and smaller α-crystalline grains. However, as the BHET content increases, the introduction of the BHET chain segments disrupts the regularity of the PA6 molecular chain. This disruption hinders the crystallization process of the α-crystalline form and slows down the growth rate of the grains. Although the growth rate is slow, the relatively long crystallization time allows more time for the grains to grow, leading to an increase in grain size. At the same time, the proportion of α-crystalline forms in the copolymer decreases, resulting in the formation of more γ-crystalline grains. These grains have limited space to grow, resulting in a gradual decrease in the γ-crystalline grain size.

**Figure 6:**
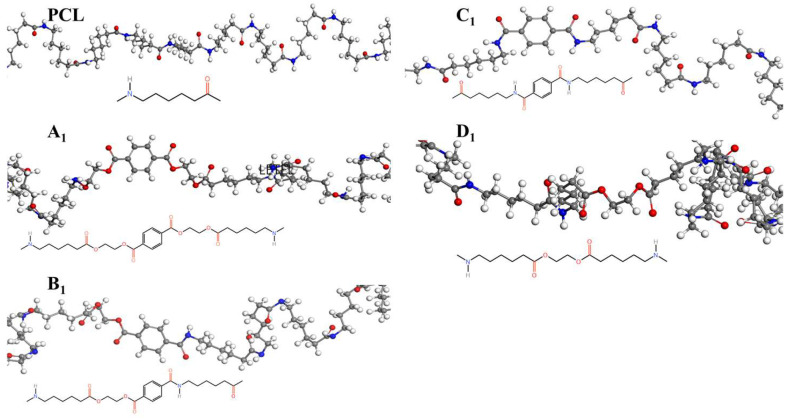
Molecular chain modeling of P(CL-*co*-BHET) copolymers.

**Figure 7:**
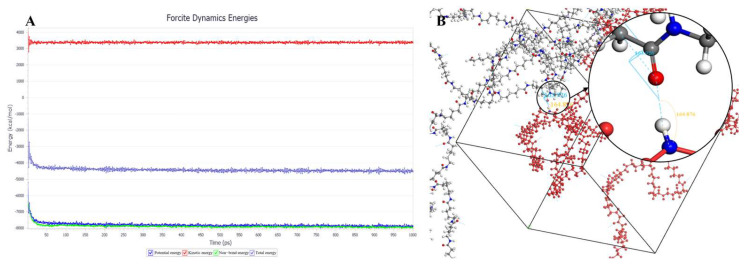
(**A**): Results of energy optimization of P(CL) chains in 3D periodic cells in molecular dynamics calculations; (**B**): simulation results of hydrogen bond formation of P(CL) chains in 3D periodic cells.

**Figure 8:**
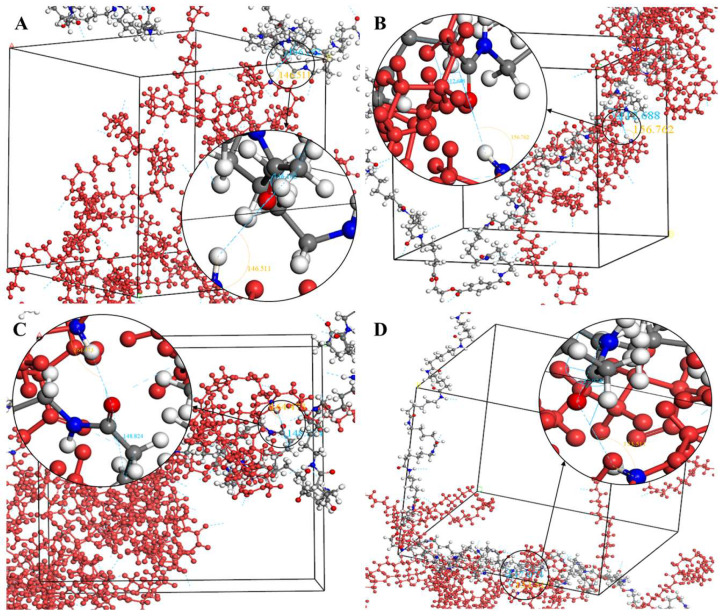
(**A**): Simulation results of hydrogen bond formation between a P(CL) chain and an A_1_ chain in a 3D periodic cell; (**B**): simulation results of hydrogen bond formation between a P(CL) chain and a B_1_ chain in a 3D periodic cell; (**C**): simulation results of hydrogen bond formation between a P(CL) chain and a C_1_ chain in a 3D periodic cell; (**D**): simulation results of hydrogen bond formation between a P(CL) chain and a D_1_ chain in a 3D periodic cell.

The crystallization behavior of the P(CL-*co*-BHET) copolymers was simulated using the Materials Studio (MS) software. Initially, the molecular chains of pure PA6 and P(CL-*co*-BHET) copolymers were modeled. The pure PA6 molecular chain consisted of 100 caprolactam repeating units (e.g., Figure 6 P(CL) chain). Due to ester–amide exchange, the distribution of the BHET chain segments in the molecular chain could result in four possible configurations (A, B, C, and D), as shown in Table 1. Therefore, four distinct structures were modeled (A_1_, B_1_, C_1_, and D_1_), each containing 36 caprolactam repeating units and 1 BHET structural unit. This configuration ensured that the mass fraction of BHET was approximately 5%, aligning closely with the experimental value. Taking P(CL) as an example, two P(CL) molecular chains were placed into a 3D periodic cell for structural optimization. The size of the 3D periodic cell was 37.3 Å × 37.3 Å × 37.3 Å. Figure 7A shows the optimization results at 300 K. After 1000 ps, the energy has already stabilized. The optimized density is 1.109 g/cm^3^, which is close to the experimental density of PA6 (1.13 g/cm^3^). Figure 7B shows the simulation results in the 3D periodic cell, with one P(CL) molecular chain in red and the other P(CL) molecular chain depicted with gray C atoms, white H atoms, red O atoms, and blue N atoms. The N-H┄C=O angle was found to be 164.8°, and the dihedral angle formed by the hydrogen bond to the main chain was 163.9°. Figure 8 shows the results of the crystallization simulation of the P(CL) molecular chain with the A_1_, B_1_, C_1_, and D_1_ molecular chains in a 3D periodic cell. The detailed data are summarized in Table 5. The number of hydrogen bonds is determined from the average counts over the last 800 steps of the optimization process, which tends to stabilize. Compared to hydrogen bonds formed by two P(CL) chains, the addition of BHET in the four structures resulted in a slight decrease in the N-H┄C=O angle, a significant reduction in the dihedral angle of hydrogen bonds with the backbone, and fewer hydrogen bonds. This is likely due to the BHET repeating unit altering the molecular chain structure, changing interactions between neighboring atoms, and disrupting the regularity of hydrogen bonding, thus affecting the crystalline shape. In the previous study on the crystallization mechanism of nylon by Siddharth et al. [26], simulations on the α and γ-crystalline forms of PA6 showed that while the N-H┄C=O angles did not differ significantly, the dihedral angle formed by hydrogen bonding with the main chain in the γ-crystalline form was significantly reduced compared to that in the α-crystalline form. The introduction of BHET changed the geometrical configuration of hydrogen bonding between molecular chains, altering the directionality of hydrogen bonding and significantly impacting the spatial conformation of the molecular chains. This change affected the stacking of molecular chains during crystallization, causing the crystalline type of PA6 to shift from α-crystalline to γ-crystalline and impeding the formation of hydrogen bonds, leading to a significant decrease in their number. This aligns with the experimental observations of a crystalline phase transition, decreased melting point, reduced crystallinity, and increased crystallization temperature in P(CL-*co*-BHET) copolymers.

## 4. Conclusions

In this study, P(CL-*co*-BHET) copolymers were synthesized, and the interplay between the molecular structure, hydrogen bonding changes, thermal properties, and crystalline behavior of the copolymers was explored. The incorporation of BHET segments affects the regularity of hydrogen bonding, leading to a slight decrease in the N-H┄C=O angle, a marked reduction in the dihedral angle formed by hydrogen bonds with the main chain, and a significant decrease in the number of hydrogen bonds. These changes result in a lower melting point, reduce thermal stability, and cause a gradual shift in the crystalline type from α-crystalline to γ-crystalline. The grain size of the α-crystalline form gradually increases until it disappears, while the grain size of the γ-crystalline form decreases. This study provides valuable insights into the crystallization behavior of polyester amides and offers a foundation for predicting and tailoring the properties of polyester amide polymers. These findings highlight the potential of P(CL-*co*-BHET) copolymers for applications in fields where adjustable thermal properties and crystalline structures are desired, such as in the development of low-melting-point hot-melt adhesive materials and biodegradable plastics. Future work will focus on exploring the mechanical properties and long-term stability of these copolymers to further assess their suitability for practical applications.

## Figures and Tables

**Table 1:** Possible structures of P(CL-*co*-BHET) copolymers and corresponding atom numbering.

Serial Number	Structures and Atom Numbering
P(CL)	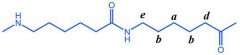
A	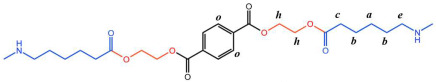
B	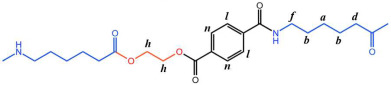
C	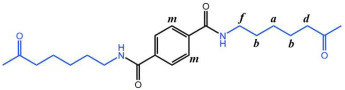
D	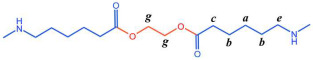
E	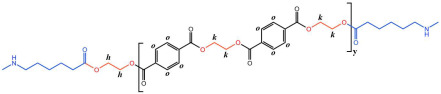
F	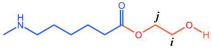

**Table 2:** The thermal properties of the PA6 and P(CL-*co*-BHET) copolymers.

Sample	Melting Point (°C)	Crystallization Temperature (°C)	Crystallinity (%)
PA6	218	166	25.4
P(CL-*co*-BHET = 100/5)	204	163	25.4
P(CL-*co*-BHET = 100/15)	195	148	20.1
P(CL-*co*-BHET = 100/30)	175/182	120	15.5

**Table 3:** The thermal stability properties of the PA6 and P(CL-*co*-BHET) copolymers.

Sample	T_d5%_ (°C)	T_f1_ (°C)	W_1_ (%)	T_f2_ (°C)	W_2_ (%)
PA6	386	N/A	N/A	452	35.5
P(CL/BHET = 100/5)	347	401	67.7	448	27.6
P(CL/BHET = 100/15)	339	384	64.2	450	19.4
P(CL/BHET = 100/30)	315	373	48.5	450	10.0

**Table 4:** The crystallographic parameters of the PA6 and P(CL-*co*-BHET) copolymers.

Sample	FWHM/°			2θ/°			*L_HKL_*/nm		
	α_1_	γ	α_2_	α_1_	γ	α_2_	α_1_	γ	α_2_
PA6	1.13	N/A	1.43	20.3	N/A	23.8	7.06	N/A	5.61
P(CL/BHET = 100/5)	0.36	0.9	0.5	20.2	21.5	23.9	22.2	8.88	16.1
P(CL/BHET = 100/15)	0.12	1.1	0.1	20	21.5	23.6	66.4	7.27	80.2
P(CL/BHET = 100/30)	N/A	1.9	N/A	N/A	21.5	N/A	N/A	4.21	N/A

**Table 5:** Geometric properties and number of hydrogen bonds obtained from simulations.

Mold	Angle N-H┄C=O (°)	Number of Hydrogen Bonds	φ [(NH)-(CO)-(CH_2_)-(CH_2_)] ^a^ (°)
P(CL) + P(CL)	164.8	121	163.9
P(CL) + A_1_	146.5	75	146.2
P(CL) + B_1_	156.8	80	112.7
P(CL) + C_1_	164.4	80	148.8
P(CL) + D_1_	133.5	84	147.1

^a^ dihedral angle formed by interchain hydrogen bonding with the main chain of a molecular chain containing the BHET structural unit -CH_2_-CH_2_-.

## Data Availability

The authors will supply the relevant data in response to reasonable requests.
